# Gelatin Matrix as Functional Biomaterial for Immobilization of Nanoparticles of Metal-Containing Compounds

**DOI:** 10.3390/jfb14020092

**Published:** 2023-02-08

**Authors:** Oleg V. Mikhailov

**Affiliations:** Department of Analytical Chemistry, Certification and Quality Management, Kazan National Research Technological University, 420015 Kazan, Russia; olegmkhlv@gmail.com

**Keywords:** functional biomaterial, gelatin, metal-containing compound, metal complex, immobilization, nanoparticles

## Abstract

The data concerning the synthesis and physicochemical characteristics of specific functional biomaterials—biopolymer-immobilized matrix systems based on gelatin as an array and chemical compounds, which include atoms of various metal elements—are systematized and discussed. The features of this biopolymer which determine the specific properties of the immobilized matrix systems formed by it and their reactivity, are noted. Data on gelatin-immobilized systems in which immobilized substances are elemental metals and coordination compounds formed as a result of redox processes, nucleophilic/electrophilic substitution reactions, and self-assembly (template synthesis), are presented. The possibilities of the practical use of metal-containing gelatin-immobilized systems are promising for the future; in particular, their potential in medicine and pharmacology as a vehicle for “targeted” drug delivery to various internal organs/tissues of the body, and, also, as potential biosensors is noted.

## 1. Introduction

The history of the immobilization of nanoparticles in a gelatin matrix actually began with the advent of a special method that dominated for a long time among the means of recording information—the silver halide photographic process—the final result of which was a photographic image consisting of elemental silver [1,2]. The source materials for this were the so-called silver halide photographic materials, which were micro- and/or nanoparticles of various silver halides AgHal (Hal = Cl, Br, I, the ratio between which varied depending on the type of photographic material) uniformly dispersed in a gelatinous massif, deposited in the form of thin films on a substrate (usually glass or polymer). These objects were in fact nothing more than silver halide gelatin-immobilized matrix systems. Even in the second half of the 19th century, the high sensitivity of silver halides to visible light was discovered [3]. That is why AgHal began to play the role of an agent in the processes of information registration, sensitive to various quanta of electromagnetic radiation—from infrared to X-rays and gamma radiation—with gelatin films performing the role of binder. As a result of the impact of radiation quanta on such systems (the so-called exposure), so-called latent image centers consisting of nanoparticles of elemental silver, as well as nanoparticles of other compounds that could be formed as a result of the interaction of chemical compounds of noble metals (gold, platinum, etc.) specially introduced into silver halide films (the so-called sensitizers) with gelatin molecules. When exposed silver halide photographic materials were treated with special solutions containing inorganic and/or organic substances with a pronounced electron-donor function (the so-called reducing donors), collectively called “developers”, silver halides were reduced to elemental silver according to the scheme AgHal + e → Ag + Hal^−^, which occurred at the latent image centers at a much higher (by at least three orders of magnitude) rate than outside these centers. Thus, these centers were nothing more than a kind of catalyst for the reduction of AgHal. In this regard, it should be specially noted that this catalytic process could only occur in a gelatin array. Numerous attempts to replace gelatin with some other binder, both natural and synthetic, have invariably failed. As a result of this process, elemental silver was formed in the gelatin layer of the AgHal photographic material, which served as the “working material” for constructing a photographic image. That part of AgHal, which did not enter into the catalytic process, was removed from the photo layer at the second stage by the action of the so-called fixing solutions containing substances that form stable and well-soluble silver compounds in water [1,2]. Initially, these were solutions containing sodium or potassium cyanide, later sodium thiosulfate. The resulting photographic image, as a rule, was grey or black (depending on the concentration of elemental silver per unit volume of the photographic layer), as a result of which it was called a “black and white image”, and the process of obtaining it, the “black-white photographic process” or, more simply, “black-white photography”. In such an image, however, the dark and light places in the photographed object seemed to change places, since the greater the brightness of the area of this object, the darker it turned out to be in the black and white image; the so-called negative was formed. Using it, it was possible to obtain a positive by exposing it to another silver halide photographic material, and then processing the exposed photographic material, first in developing, and then in fixing solutions. These images were the first gelatin-immobilized matrices containing either elemental metals or chemical compounds containing metal chemical elements as immobilized substances. Later, using the ideas of black and white photography, a more complex version of the silver halide photographic process was created—color silver halide photography. In this process, a color image, the “working material” for which various organic dyes served, was obtained [1,2,4,5,6]. However, this process was much more labor-intensive than black and white photography, and also required a much more highly skilled performer. This circumstance to some extent contributed to the emergence of a certain intermediate variant, which can be called the “monochrome silver halide photo-process”, since as a result of its implementation, not a polychrome photographic image, as in the case of a color photograph, was obtained, but a monochrome one, having a certain color shade—blue, green, brown, etc. This option, also called “toning”, assumed the additional processing of the original silver image, as a result of which the elemental silver contained in it was transformed to some degree or another into some other insoluble compound of it (usually, into a silver halide), with the simultaneous formation of some intensely colored compound containing another chemical element—a metal (nickel, copper, cobalt, etc.). Thus, gelatin-immobilized matrices containing, along with elemental silver, other chemical compounds, were obtained [7,8,9].

In principle, there are two main approaches to the immobilization of a substance in a gelatin matrix:

- Dispersion of previously created particles of a given substance in an array or a thin layer of gelatin;
- The formation of particles of a given substance directly in a gelatin mass or a thin gelatin layer as a result of chemical transformations occurring with the participation of any other gelatin-immobilized chemicals.

In practice, depending on the scope of the application, both of these options were involved in one way or another. In particular, within the framework of the silver halide photo process mentioned above, the first of them was used in the manufacture of the AgHal photographic materials themselves, and the second was used in the processes occurring as a result of the formation of various photographic black and white (silver) and color (mono- or polychrome) photographic images, and namely the variant that provides for additional processing of an image consisting of elemental silver served as the ideological basis for the creation of a very significant range of metal-containing gelatin-immobilized matrix systems, which will be the key objects of this review article. A special place among these objects is occupied by gelatin-immobilized matrices containing complexes of various d-elements, primarily with chelate and macrocyclic organic compounds. They are interesting not only from a purely academic point of view, but also from a practical point of view, since, substances immobilized in them consist of nanoparticles, and, in principle, they can be used in various branches of engineering and technology. One such promising application is their use as components for the production of functional biomaterials, which, in turn, can be in demand in biotechnology, pharmacology, medicine, and related industries. The given review article is written in order to draw close attention to these exotic objects of precisely those researchers who work specifically in the field of so-called “life sciences”—biotechnology, pharmacology and medicine, and who can read articles in such a highly authoritative scientific journal as is the *Journal of Functional Biomaterials*. However, before considering them, it is necessary, in our opinion, to provide certain information about that unique substance without which such systems could not exist, namely, about gelatin, and upon which the immobilization of nanoparticles in the second of the above options is dependent.

## 2. Physicochemistry of Gelatin as a High-Molecular Component of Immobilized Matrix Systems

**Figure 1:**
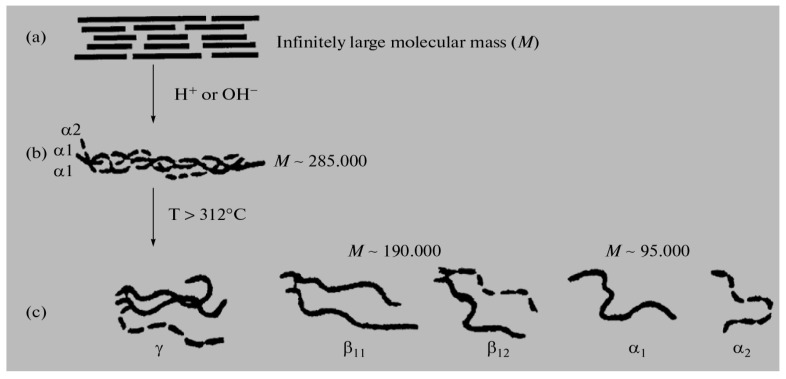
Different levels of organization of collagen and gelatin: (**a**) the area of connective tissue collagen with strong cross-linking, (**b**) a separate molecular fragment of collagen/gelatin, consisting of three chains; (**c**) three kinds of formations of gelatin molecules in the form of balls, resulting from the destruction of collagen. (Reproduced with permission from Springer Nature).

**Figure 2:**
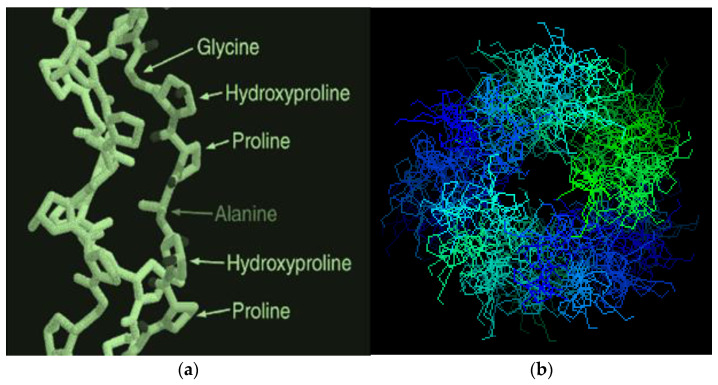
Schematic image of three polypeptide α-chains forming a triple-helix of gelatin (**a**); coils and nets in the gelatin structure (**b**). (Reproduced with permission from Elsevier).

Gelatin is a mixture of different fractions each of which having general formula I, and consists of amino acid residues interconnected by peptide bonds (where R_1_, R_i_, R_j_, R_k_ are 
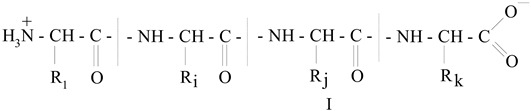
various radicals). The molecular mass (*M*) of these fractions is in a very wide range, from 15,000 to ~300,000 Daltons, so among them there are both low-molecular weight and high-molecular weight polypeptides [10,11,12,13,14,15]. In general, gelatin contains residues of 18 natural amino acids out of 20, with the exception of cystine and cysteine. Gelatin is often called a “biopolymer”, and although such a name is not entirely correct (since the concept of “polymer” in general and “biopolymer” in particular includes only those synthetic and natural high-molecular compounds for which a regularly repeating structural unit can be indicated, as, for example, in polypropylene (–CH_2_–CH_2_–CH_2_−)_n_ or in natural rubber (–CH_2_–C(CH_3_)=CH_2_−)_n_, which is not the case for gelatin), we will also use this term from time to time. Gelatin is obtained from another natural protein that is part of various connective tissues of animals, namely collagen, by exposing it to either sulfuric, hydrochloric, orthophosphoric acids (the so-called acid gelatin) or an aqueous solution of calcium hydroxide (the so-called alkaline gelatin). In this regard, three levels of organization of polypeptide fractions associated with it and gelatin are distinguished (Figure 1). The structure and properties of both of these biopolymers have been repeatedly studied over the past few decades (see, in particular [14,15,16,17,18,19,20,21,22,23,24,25,26,27,28,29,30,31,32,33,34,35,36,37,38,39,40,41,42,43,44,45,46,47,48,49,50,51,52,53,54,55,56,57,58,59,60,61,62,63,64,65,66,67,68,69,70]). The total number of works on this topic is at least several hundred, and in this article, it is not possible even to simply cite them in full. As a result of the studies, it was found that the macromolecules of these high-molecular polypeptides consist of three polypeptide chains with almost the same molecular mass (Figure 2a); moreover, two of them, as a rule, are almost identical to each other in the set and sequence of amino acids (the so-called α1-chains), while the third (the so-called α2-chain) in this respect differs from the two others [26,32,33]. Each of these chains contains over 1000 amino acid residues [19,50] and has *M* ~ 95,000 Daltons. The gelatin molecule is stabilized by the formation of covalent cross-links, both within the gelatin triple helix and between gelatin helices. During the transformation of collagen into gelatin, a polydisperse mixture is formed containing single (α_1_ and α_2_), double (β_11_ and β_12_) and triple (γ) polypeptide chains, which, unlike collagen macromolecules, are formed in the form of coils or clots (Figure 2b).

In [14,17], two alternative gelatin molecule structures, in each of which the formation of triple helices was postulated, were proposed. Both of these structures consist of three parallel polypeptide α-chains twisted into a left-handed helix. At the same time, the helical chains in them are stabilized by interchain hydrogen bonds –(–)NH….O=C(–)–. However, in the structure proposed in [14], only one bond is required for every three amino acid residues, while in the structure of [17], two. An unambiguous choice between these two alternative structures, interestingly, turned out to be impossible even after X-ray diffraction analysis. Subsequently, various independent physicochemical methods of analysis were repeatedly proposed to solve this problem, but, despite this, the question of the exact structure of gelatin in connection with the above theoretically permissible possibility remains open to this day. Each of the peptide fragments of the gelatin molecule is characterized by the conjugation of the π-electrons of the C, N, and O atoms, as a result of which the (–C–C–NH–C(=O)–) group acquires a quasi-planar structure. The carbon–nitrogen interatomic distance in this structural fragment is 132 pm, which is much shorter than the C–N single bond length (147 pm), so this bond is very close to a double bond in terms of the degree of multiplicity.

Already more than 50 years ago [23], it was established by means of electron microscopy that the diameter of gelatin macromolecules at the level of organization (b) (Figure 1) is 1400 pm, while their length is 285,000 pm. These values are in good agreement both with similar data found in even earlier work [16] on measurements of the light scattering and viscosity of gelatin solutions and with later data presented in works published in the 21st century [33,34,35,36,37,38,39,40,41,42,43,44,45,46,47,48,49,50]. In view of the foregoing, it can be argued that the molecules of various fractions of gelatin, as well as collagen molecules, are sharply asymmetric and anisometric. On the other hand, which is characteristic, their width is more than an order of magnitude smaller than their length; taking into account the fact that both very strong intramolecular and intermolecular interactions take place for these molecules, one can theoretically expect that, under certain conditions, a liquid–crystal state can be realized in gelatin arrays.

**Figure 3:**
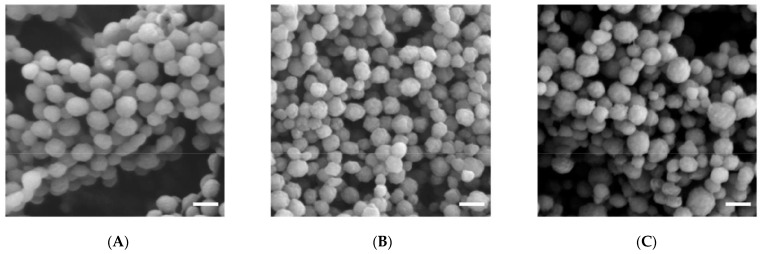
SEM images of lyophilized gelatin nanoparticles with different surface charges. (**A**) Zeta potential (19.0 ± 0.8), linear size (128 ± 18) nm; (**B**) zeta potential (2.9 ± 0.3), linear size (104 ± 23) nm; (**C**) zeta potential (–11.6 ± 0.8), linear size (133 ± 43) nm. (This figure was made by the author of the given article according to the data presented in [71]).

Gelatin arrays contain many endless networks formed by long rows of molecules interconnected by a limited number of cross-links [30,32,33], due to this, they have a high degree of flexibility and elasticity. Such an internal structure is potentially already a priori convenient for the immobilization of a wide variety of substances with their fixation due to intermolecular interactions; on the one hand, it does not allow any rigid crystalline blocks to be realized, on the other hand, it has a sufficiently large number of cells for the receiving and subsequent “fixing” of molecules of the immobilized substance. Even being already filled with molecules of any chemical compound, these cells retain a certain freedom of movement in space. The size of such a cell was once estimated in [51]. According to the data of this work, the average linear size of such a cell, assuming a spherical shape for it, is (8.91–10.22) nm, and assuming a cubic shape, it is (7.18–8.24) nm. As can be seen from these values, with similar cell sizes, it is possible to introduce into it both rather large molecules of the immobilized substance and nanoparticles of various substances. Thus, if the reaction of the formation of any substance proceeds in the gelatin matrix, the probability of its immobilization in the array (as well as in a thin layer) of this biopolymer seems to be quite high. In this regard, it is noteworthy that gelatin itself quite easily forms nanoparticles, both in the case when its molecules are neutral and in cases when they carry a positive or negative charge [71]. Images of these nanoparticles are shown in Figure 3.

As for the chemical properties of gelatin, it is in principle possible for it to participate in each of the three main types of chemical reactions occurring in solutions or in the solid phase, namely, acid-base reactions, redox processes, and complex formation processes. According to the Brønsted–Lowry proteolytic theory [72,73,74], it is a typical ampholyte, since it contains acids (β-carboxylic aspartic acid, β-carboxylic glutamic acid, phenol-hydroxyl tyrosine) and basic (β-imidazole histidine, **ε**-amine lysine, δ-guanidine arginine) groups. Gelatin is an electrically neutral compound, but in reality, it exists in aqueous solutions in a zwitterionic form. Therefore it is quite natural that it has a so-called isoelectric (isoionic) point (p*I*), in which the positive charges of the terminal NH_3_^+^ groups are neutralized by the negative charges of the terminal COO^–^ groups. At the same time, the so-called alkaline gelatin p*I* is in the range pH = 4.8–5.1; it is characteristic that at the pH value corresponding to this point, all the main groups carry a positive charge and the gelatin molecules contain the same negative charges due to the deprotonation of most (though not all) carboxyl groups. For the so-called acid gelatin, the range of possible pI values is much larger (7.0–9.5); in this case, all carboxyl groups are deprotonated and their charge is balanced by the positive charge of guanidine and most **ε**-amino groups. It is noteworthy that the p*I* value for the “progenitor” of gelatin, collagen (9.0–9.5), also falls into the same range. This implies the following very important circumstance: all chemical processes leading to the immobilization of chemical compounds in a gelatinous array proceed if gelatin macromolecules have an electric charge (+), if the pH value of the reaction medium (actually determined by the pH value of the solution in contact with the biopolymer matrix) is lower than the value of p*I*, and (–) at the inverse ratio of the indicated parameters. Since gelatin contains peptide groups, as well as amino acids containing the so-called labile sulfur (in the form of thiol groups), it can also act as a reducing agent in redox reactions, in particular, reduce noble metal ions (Au^3+^, Pt^4+^) to ions of the same metals with lower charges or even to corresponding elemental metals. Gelatin also has the ability to act as a polydentate chelating ligand in complex formation processes. This property is connected with the presence of peptide groups (–C–NH–C(=O)–C–) in its molecular structure. Complexing processes with the participation of gelatin molecules have very complex and ambiguous characters since it is possible to form both homoligand metal complexes (containing only one molecule of a given protein as a ligand) and heteroligand ones containing two or even more different proteins in the inner coordination sphere. In this connection, it should be noted that the complexing of gelatin molecules with some cations, in particular Cr(III), Ti(IV) and Zr(IV), underlies a practically important process called “mineral tanning”. As a result of this process, there is a “crosslinking” of the polypeptide fragments of this biopolymer taking place. Owing to it, in particular, a significant increase in the viscosity of water-gelatin solutions, an increase in its transition temperature from a solid to a liquid state (which is visually perceived as a kind of “melting”, although this term is incorrect here since gelatin is an amorphous substance), as well as increasing the mechanical strength of the gelatin layers, are observed. It should be noted in this connection that the above “crosslinking” of polypeptide fragments can also occur when exposed to a number of organic substances, such as aldehydes, triazines, and epoxides [1,2]. This process is described in detail in the review [75], as well as in the above-cited monographs [1,2] devoted to silver halide photography.

## 3. Specific Features of Immobilization in a Gelatin Matrix

The relatively large gaps between the chains of the spatial network in the molecular structure of gelatin allow molecules and ions of low-molecular substances, unlike large colloidal particles or macromolecules, to diffuse into its intermolecular hollows almost as easily as into liquid-phase solvents. At the same time, gelatin layers have high transparency and plasticity, which makes them very convenient for studying by various spectroscopic methods (primarily spectroscopy using the UV, visible, and near-IR spectral regions). It is also important that gelatin arrays are quite easily destroyed under the influence of various proteolytic enzymes (trypsin, *Bacillus mesentericus*, *Bacillus subtilis*, etc.). Due to this, chemical compounds immobilized in it can be isolated from it in the form of solid phases without any special problems and analyzed by the same modern physicochemical methods as solid substances isolated from liquid-phase or gas-phase reaction systems. Finally, gelatin is well known to be a hydrophilic substance. This circumstance also seems to be important for the realization of various processes in it, since most chemical reactions occur precisely in aqueous solutions.

One more circumstance that is very important for understanding the specifics of reactions in gelatin-immobilized matrix systems should be specially noted. As mentioned above, due to the specific molecular structure of this biopolymer, it has many nanoscale intermolecular hollows, in which molecules of various chemical compounds and/or their aggregates (associates) can be fixed with sufficiently high rigidity. The possibility of their movement outside the hollows in which they are located is very limited, and therefore, their interaction with other chemical compounds contained in solutions in contact with the gelatin matrix will occur within these hollows. Thus, the indicated hollows can be considered as a kind of molecular nanoreactors, and the gelatin matrices, in which they are located, as organizing systems. Within such systems, there is a “forced” decrease in entropy compared to the entropy of a system in which the same substances are in solution or solid phase. Such a “forced” decrease in entropy, it is easy to see, leads to the fact that, in the framework of the well-known Gibbs–Helmholtz Equation (1).

*Δ*G(*T*)= *ΔH*^0^ − *TΔS*^0^ (1)

(*ΔH*^0^ and *ΔS*^0^ are the standard enthalpy and entropy of the reaction, respectively, *ΔG(T)* is the change in the Gibbs energy as a result of the same reaction, and *T* is the absolute temperature). The tangent of the slope of the linear dependence *ΔG(T)* to the abscissa axis decreases, and therefore the temperature range *T* in which this chemical reaction is thermodynamically permitted increases. Indeed, since entropy is an additive quantity, then in such an organizing system for this thermodynamic parameter there will be an Expression (2)

*ΔS*^0^ = *ΔS*^0^′ + *ΔS_os_* (2)

where *ΔS*^0^ is the standard entropy of the reaction in the absence of the above “forced” entropy reduction, *ΔS*^0^′ is the standard entropy of the reaction in the organizing system, and *ΔS_os_* is the entropy change determined by the specifics of the given organizing system. As a result, the dependence equation *ΔG′(T)* in a system where such a “forced” decrease in entropy takes place will be written as (3)

*ΔG′(T)* = *ΔH*^0^ − *TΔS*^0^′ = *ΔH*^0^ − *T*(*ΔS*^0^ − *ΔS_os_*) (3)

and, as it is easy to note, relation (4) will take place

(*ΔS*^0^ − *ΔS_os_*) < *ΔS*^0^ (4)

**Figure 4:**
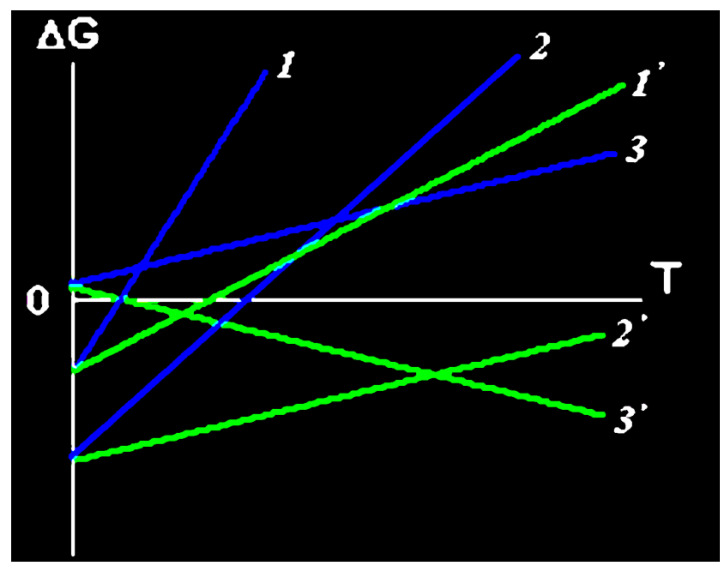
*ΔG*(*T*) dependences (*1*, *2*, *3*) and *ΔG′*(*T)* ones (*1′*, *2′*, *3′*) for three various variants of chemical processes: *1*, *1′* and *2*, *2′*—for processes having *ΔH* < 0 which, in principle, can be realized in the reactionary systems without “compulsory” decrease in entropy indicated, *3*, *3′*—for reactionary process having *ΔH* > 0 which can be realized only in the case of “compulsory” decrease in entropy. Lower slope of straight lines of *ΔG*(*T*) and *ΔG′*(*T*) dependences to abscissa axis is clearly seen. (Reproduced with permission from Elsevier).

This circumstance is illustrated in Figure 4. In the case of reactions proceeding with a decrease in entropy (as, for example, in the processes of the so-called “self-assembly” of molecules from simpler compounds), it acquires special significance, since a significant part of such reactions at those relatively low temperatures, at which they are thermodynamically allowed, proceeds at a very low rate, and at high rates.

The general problems related to immobilization in polymer arrays and reactions involving immobilized chemical compounds are considered in monographs and review articles [76,77,78,79,80,81,82,83]. In principle, there are two options for the immobilization of a substance at the molecular level—either with the use of functional groups of a molecule of a macro-molecular compound (polymer) and the formation of metal-polymer bonds (which can be conditionally called “chemical immobilization”) or due to dispersion, orientation, induction and other similar methods. Types of interaction, for which only the presence of a well-developed polymer surface (which can conventionally be called “physical immobilization”) is sufficient [76,77]. The second of these variants of immobilization can be carried out in a variety of ways—by dispersion, adsorption or precipitation of the target substance in the polymer layer, sputtering, impregnation, etc. In this case, the most preferable is the deposition of the target chemical compound directly in the polymer array, carried out as a result of appropriate chemical transformations, since it is in this case that the nanostructural level of organization of the immobilized substance can be achieved. Gelatin belongs to those high-molecular compounds, the molecules of which are associated with the immobilized substance almost always only due to so-called physical immobilization [83] (although, of course, chemical immobilization can also take place in it, albeit to an insignificant degree).

## 4. Immobilization of Nanoparticles of Elemental Metals in a Gelatin Matrix Using Redox Reactions

**Figure 5:**
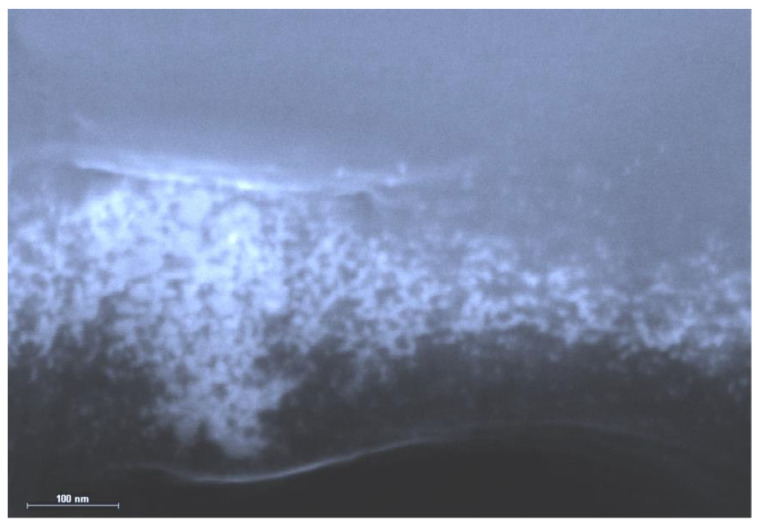
SEM elemental silver particles contained in the Ag-gelatin-immobilized matrix after the procedure of “re-precipitation”. (This figure was made by the author of the given article according to the data presented in his publication [87]). (Reproduced with permission from Elsevier).

As already mentioned in the Introduction, historically, the earliest of the gelatin-immobilized matrix systems containing elemental metals were silver-containing matrix implants obtained from silver halide photographic materials by reducing silver halides (AgHal) under the influence of specific reducing solutions (developers). During the period of active development of silver halide photography, many works were devoted to the study of the processes of their formation, the number of which is measured in many thousands; at the same time, a wide variety of chemicals were used as reducing agents for silver halides in these developers—both organic (in particular, metol, hydroquinone, amidol) and inorganic (for example, sulfates or chlorides of Fe(II), Cr(II), Ti(III), V(III)). The particles of gelatin-immobilized elemental silver contained in the matrices obtained in this way were also microsized, but depending on the nature of the halide ions, the size and shape of AgHal microcrystals, as a rule, they imparted to the gelatin layer of the matrix either a black color or a black color with a weak but pronounced blue or brown tint [1,2]. It was possible to obtain elemental silver nanoparticles only as a result of some processes so-called “reprecipitation” of elemental silver, in which the previously obtained gelatin-immobilized elemental silver was subjected to a two-stage process, during the first stage of which, under the influence of some oxidizing agent, it was converted into a sparingly soluble compound of this element (as a rule, into silver bromide AgBr), and at the second stage, was subjected to reduction to elemental silver using a strong reducing agent, tin dichloride, SnCl_2_. The details of elemental silver “re-precipitation” using such a process are described in [84,85,86,87,88]. Similar processes were also considered in [89,90,91,92,93,94,95] using other chemical compounds as reducing agents. In this regard, it should be noted that the processes described in [84,85,86,87,88], according to the data of XRD analysis [86,87], gave rise to a specific phase of elemental silver, which imparted a red-brown color to the gelatin layer. Although a priori, taking into account everything said in Section 3, one can expect that elemental silver nanoparticles should be formed as a result of the “re-precipitation” process; experimental evidence for this was obtained only a few years ago using SEM [87] (see Figure 5).

Information about the immobilization of other elemental metals in the gelatin matrix at the present time has rather a peculiar character. On the one hand, it cannot be said that no research has been carried out in this direction because publications devoted to it still exist, and there are quite a few of them; this can be confirmed by the review article [96], which contains more than 200 references to such publications. However, firstly, this article itself was published more than 40 years ago, and secondly, almost all the publications cited in it, as well as the review [96] itself, are not available on the Internet. In connection with this, it should be said that all these publications are not related to the issue to which our review is devoted, but to one that in our time has only a purely historical interest, namely, silver halide photography (which has now been practically supplanted by other, more effective ways of recording information, and above all digital technologies). At the end of the 1970s of the 20th century in the industry that was associated with the production of silver halide photographic materials, due to the shortage of silver that had already begun at that time, the problem of all-round saving of this precious metal arose with all acuteness. This problem was exacerbated by the circumstance that the photographic industry at that time consumed almost 30% of all silver mined in the world. This circumstance has made popular photographic processes on silver halide photographic materials (in fact, as mentioned above, silver halide gelatin-immobilized matrices) aim to reduce the content or even completely replace this precious raw material in photographic images obtained as part of a black and white photographic process. In this connection, processes with so-called “physical development”, gained some popularity. The idea of these processes was that the three functions that AgHal performs in black and white photography, namely (a) a photosensitive compound, (b) a compound from which centers of development are formed, (c) “building material” for forming a photographic image, are distributed between two or even three different chemical compounds. In general, the functions (a–c) can be represented by stylized Schemes (5), (6) and (7), respectively.

A + hν → B(5)

B + C → D(6)

M^z+^ + Red → M + Ox (7)

where A is a light-sensitive compound, B is a product formed by the action of radiation on A, C is a substance that forms catalyst D upon reaction with substance B, M^z+^ is a reducible metal ion, M is a product of M^z+^ reduction, Red and Ox are a reducing agent and its product oxidation, respectively [96]. In this case, reaction (7) must be catalyzed by both substance D and elemental metal M, i.e., be autocatalytic. Using this idea, photographic materials were created, and the content of AgHal (and, accordingly, silver) was either much lower than in traditional silver halide photographic materials (since here AgHal is needed only for the formation of the so-called catalytic centers containing elemental silver, on which there is a process of reduction of some other metal-containing compound) or in general, it was absent from them altogether. To implement this process in gelatin-immobilized matrices, various reactions of chemical precipitation of metals from aqueous solutions were used, which in themselves are kinetically retarded and proceed at a low rate in the absence of an appropriate catalyst, but if they are present in the gelatin layer of the matrix, their rate increases sharply. In this connection, first of all, it should be noted the preparation of gelatin-immobilized matrix systems containing elemental nickel and elemental copper. In the first case, the immobilized substance was obtained as a result of the reduction of nickel(II) chloride or sulfate with sodium hypophosphite NaH_2_PO_2_ according to the Scheme (8)

2HPO_2^−^_ + Ni^2+^ + 2OH^−^ → Ni + H_2_ + 2HPO_3^−^_ (8)

and in the second, as a result of the process of reducing chloride or sulfate of copper (II) with formaldehyde according to the Scheme (9)

2CH_2_O + Cu^2+^ + 4OH^−^ → Cu + H_2_ + 2HCOO^−^ + 2H_2_O (9)

Reaction (8) was catalyzed only by particles of elemental palladium, while reaction (9) was catalyzed by particles of elemental copper, silver, gold, platinum, and palladium. Within the framework of the same methodological approach, gelatin-immobilized matrix systems containing some other elemental metals, namely, iron, cobalt, tin, indium, and mercury, were also obtained [96]. However, these gelatin-immobilized systems eventually attracted the attention of only those researchers who worked in the above very specific area; after it, figuratively speaking, had outlived its age, the study of such objects has actually completely stopped (at least, this is the situation at the moment).

## 5. Immobilization of Nanoparticles of Metal Complexes in Reactions of Nucleophilic Substitution and Template Synthesis

The processes associated with the immobilization of metal-containing chemical compounds in gelatin matrices using nucleophilic substitution reactions have been considered in a very significant number of original papers [97,98,99,100,101,102,103,104,105,106,107,108,109,110,111,112,113,114,115,116,117,118,119,120,121,122,123,124,125,126,127,128,129,130,131,132,133,134,135,136,137,138,139,140,141,142,143]. The theoretical foundations of the process of complex formation in gelatin-immobilized matrix systems are described in [97]. The greatest number of works in this direction were associated with the immobilization of metal complexes; in all these works, gelatin-immobilized matrices containing elemental silver were used as initial “raw materials”, which were further subjected to specific processing in three stages:

(1) Treatment of a silver-containing gelatin-immobilized matrix with an aqueous solution containing a complex of the corresponding metal ion with any hydroxy acid (oxalic, citric or tartaric), potassium hexacyanoferrate(III) (ferricyanide) K_3_[Fe(CN)_6_] and an agent for creating the necessity for the reaction of the acidity of the medium (usually slightly alkaline). As a result, elemental silver contained in the gelatin matrix was transformed into silver hexacyanoferrate(II) (ferrocyanide) Ag_4_[Fe(CN)_6_] and, simultaneously, hexacyanoferrate(II) of the corresponding metal ion was formed in the gelatin layer.

(2) Treatment of the matrix obtained at stage (1) with an aqueous solution of sodium thiosulfate (hyposulfite) Na_2_S_2_O_3_. In this case, the silver hexacyanoferrate(II) contained in the matrix was transformed into a soluble silver complex with the thiosulfate anion and was removed from the matrix into the solution in contact with it, while the metal hexacyanoferrate(II) coprecipitated with it at stage (1) remained unchanged. As a result, a metal hexacyanoferrate(II) gelatin-immobilized matrix was obtained.

(3) Treatment of the matrix obtained at the end of stages (1) and (2) with an aqueous alkaline solution containing an organic substance capable of forming a poorly water-soluble complex with a given metal ion (usually a metal chelate). This gelatin-immobilized metal chelate was the final product of the synthesis.

The details of such a process are described in [98]. Such a scheme, however, was implemented only for a small number of metal ions, namely, Ni(II), Cu(II), Fe(III), and Co(III); for other metal ions, stage (1) had to be used in two stages, namely:

(1a) Treating the silver-containing gelatin-immobilized matrix with an aqueous solution of potassium hexacyanoferrate(III) to convert elemental silver to silver hexacyanoferrate(II);

(1b) Treatment of the matrix obtained in step (1a) with an aqueous solution of chloride or bromide of the corresponding metal ion. As a result of contact of the matrix with such a solution, silver hexacyanoferrate(II) was converted into sparingly soluble silver chloride or bromide, and the metal ion was bound to the hexacyanoferrate(II) anion, turning into the corresponding hexacyanoferrate(II).

This variant of obtaining gelatin-immobilized hexacyanoferrates(II) (which, as is easy to see, were the initial metal-containing precursors for various complex formation processes) is universal and suitable for most metal ions [99]. 

Historically, the earliest of the works devoted to this topic was the publication [100], in which the immobilization of the Ni(II) chelate complex with the deprotonated form of dithiooxamide (H_2_N–C(=S)–C(=S)–NH_2_) was carried out. After that, the list of gelatin-immobilized metal complexes began to grow rather quickly. It is these that now make up the bulk of gelatin-immobilized chemical compounds in general and metal-containing compounds in particular. Initially, these were metal chelates obtained using classical complexation reactions (nucleophilic substitution), i.e., as a result of the interaction between a metal ion and any organic compound that acts as a ligand. The results of these studies are presented in [100,101,102,103,104,105,106,107,108,109,110,111,112,113,114,115,116,117,118,119,120,121,122,123]. In most cases, such metal chelates included Co, Ni, or Cu; gelatin-immobilized compounds containing any other metals were studied only in [102,108,113,114,123]. In particular, metal chelates such as Ni(II) with dimethylglyoxime H_3_C–C(=NOH)–C(=NOH)–CH_3_ and its analogs [121], Cu(II) with dithiooxamide and its substituted [103,107,111,112], Co(II) and Co(III) with 8-mercaptoquinoline [109,119,120], Fe(II) and Fe(III) with 8-mercaptoquinoline [105,113], U(VI) with 8-hydroxyquinoline and its halogen-containing derivatives [114]. At the end of the 20th/beginning of the 21st centuries for the immobilization of metal chelates, template synthesis reactions (“self-assembly”), in which the ligands contained in such metal chelates are formed as a result of the combination of simpler organic compounds in comparison with them, containing donor atoms of nitrogen, sulfur, and oxygen, were used [124,125,126,127,128,129,130,131,132,133,134,135,136,137,138,139,140,141,142,143]. In these reactions, however, only macrocyclic metal complexes, which included Co, Ni, or Cu (mainly with nitrogen-sulfur-containing chelate ligands), were obtained. It should be especially noted in this connection that in a number of cases, these reactions produce specific metal chelates, the formation of which does not take place under the traditional conditions for the implementation of these same reactions (i.e., in solution or solid phase); such a phenomenon was observed, for example, in [100,128,133,136]. This important difference between reactions occurring in gelatin-immobilized matrices and reactions under traditional conditions (in solution and solid phase) is due to two factors that we noted in Section 2 and Section 3. First: the biopolymer molecules that form these matrix systems are charged particles, which facilitate the formation of chelate-type compounds with deprotonated forms of ligands. Second: the gelatin matrix is an organizing system, due to which there is an increase in the temperature range in which the process of complex formation and/or template synthesis can be realized. A discussion of the chemistry of these processes, however, is beyond the scope of this article; more detailed information about this can be found in the original papers cited above, as well as in reviews [82,144,145,146,147,148,149,150]. In addition to obtaining gelatin-immobilized metal chelates, the possibility of the immobilization of some other metal-containing chemical compounds, namely, metal sulfides and phosphates in this biopolymer, was considered in the literature at different times, but these studies were only episodic [151,152,153].

**Figure 6:**
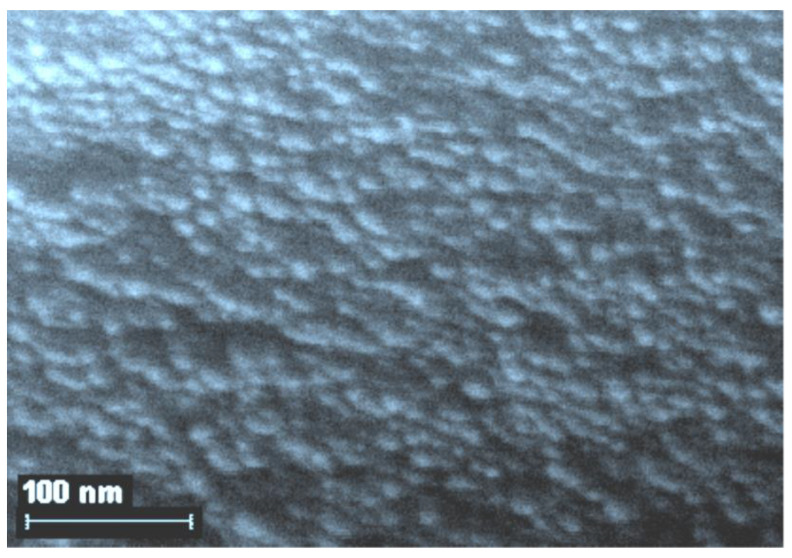
The SEM photograph of nanoparticles of gelatin-immobilized Co(II) macrocyclic metal chelate formed in a template synthesis in the GIM in the Co(II)-dithiooxamide-formaldehyde system. (This figure was made by the author of the given article according to the data presented in his publication [51]). (Reproduced with permission from Springer Nature).

**Figure 7:**
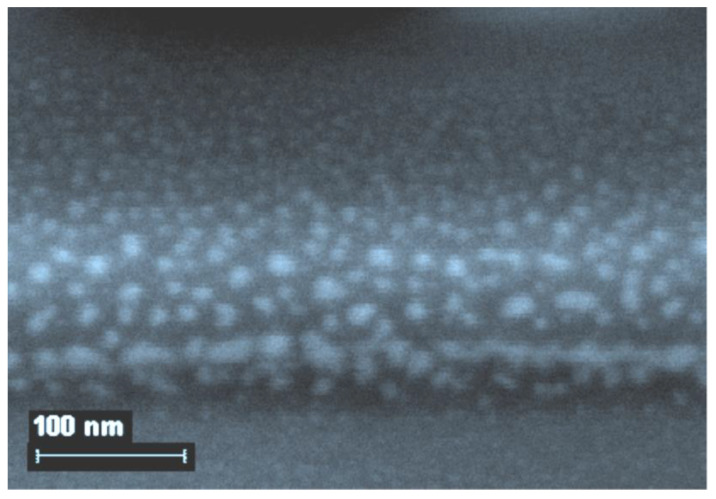
The SEM photograph of nanoparticles of gelatin-immobilized Ni(II) macrocyclic metal chelate formed in a template synthesis in the GIM in the Ni(II)-dithiooxamide-glyoxal system. (This figure was made by the author of the given article according to the data presented in his publication [51]). (Reproduced with permission from Springer Nature).

**Figure 8:**
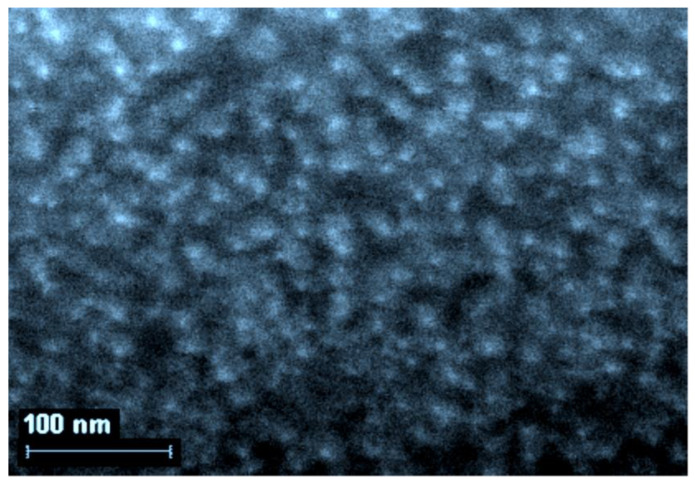
The SEM photograph of nanoparticles of gelatin-immobilized Cu(II) macrocyclic metal chelate formed in a template synthesis in the GIM in the Cu(II)-thiocarbohydrazide-acetone system. (This figure was made by the author of the given article according to the data presented in his publication [51]). (Reproduced with permission from Springer Nature).

**Figure 9:**
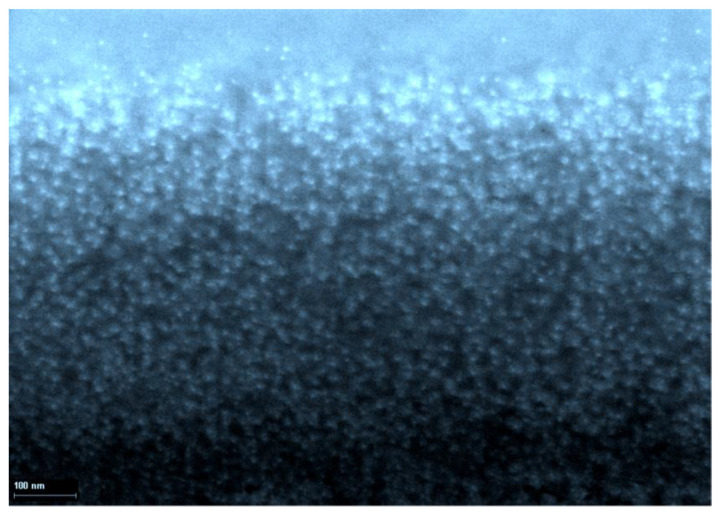
The SEM photograph of nanoparticles of gelatin-immobilized PbS. (This figure was made by the author of the given article according to the data presented in his publication [153]). (Reproduced with permission from Springer Nature).

The question of what level of organization of gelatin-immobilized metal-containing compounds in general and metal complexes in particular, remained open until the middle of the second decade of the 21st century, although taking into account the data on the internal structure of gelatin arrays, namely the sizes of hollows in its molecular structure [51], it could be expected a priori that the substances immobilized in the array of this biopolymer would mainly consist of nanoparticles. However, experimental pieces of evidence in this regard concerning gelatin-immobilized metal sulfides, elemental metals, and metal complexes appeared only in the mid-2010s; the corresponding data, namely those obtained by scanning electron microscopy (SEM), are given in the publications cited above [51,153,154], as well as in the recently published article [155]. Examples of SEM particles of metal-containing compounds formed in gelatin-immobilized matrix systems are presented in Figure 6, Figure 7, Figure 8 and Figure 9. Although data of this kind are still very limited, taking into account the specifics of the chemical compounds presented in Figure 6, Figure 7 and Figure 8 (each of which is a metal-macrocyclic and has a rather large molecular size), there is every reason to believe that gelatin-immobilized matrix systems formed as a result of those various physicochemical processes that occur in the reactions described in [124,125,126,127,128,129,130,131,132,133,134,135,136,137,138,139,140,141,142,143], will also have a nano-structural level of organization of the chemicals immobilized in them. This conclusion is all the more true for those gelatin-immobilized matrix systems that arise as a result of the implementation of the processes described in [100,101,102,103,104,105,106,107,108,109,110,111,112,113,114,115,116,117,118,119,120,121,122,123], since the size of the molecules of immobilized substances formed in these reactions, as well as their molecular masses, is much lower than those of metal macrocyclic compounds.

## 6. Immobilization of Nanoparticles of Metal Hexacyanoferrates(II) in Reactions of Nucleophilic Substitution and Ionic Exchange

Mononuclear hexacyanoferrates(II) of various metals were the very first complexes known to chemical science; in any case, potassium hexacyanoferrate(II) was obtained as early as 1704 by Disbach, and iron(III) hexacyanoferrate(II) K[Fe_2_(CN)_6_] (the so-called Prussian blue or Turnbull blue) has been known since the 13th century [156,157]. These compounds have several unique properties that make them widely used in various fields of science and engineering/technology [156,157,158]; moreover, according to the data presented, in particular, in [159,160,161,162,163] and review article [164], they can be quite easily obtained in the form of nanoparticles. On the other hand, as can be seen from Section 5, they turned out to be very successful precursors for the synthesis of very diverse metal complex gelatin-immobilized matrix systems. The general procedure for the synthesis of gelatin-immobilized mononuclear hexacyanoferrates(II) containing di-, tri-, and tetra-charged metal ions is described in [99]. These are the substances that were subsequently used to implement the processes of electrophilic substitution or ion exchange.

It has long been known that homonuclear hexacyanoferrates(II) of *d*-elements of the M_2_[Fe(CN)_6_], M_4_[Fe(CN)_6_]_3_, and M[Fe(CN)_6_] types (where M is a *d*-element atom) are capable of entering into reactions accompanied by the substitution of the M(II), M(III) or M(IV) contained in them for ions of other metals (M) [157]. Such reactions are very characteristic when M’ belongs to the category of *s*-elements. Even more than 50 years ago, as a result of such reactions, a rather significant amount of (*sd*)heteronuclear hexacyanoferrates(II) containing ions of *s*- and *d*-elements was obtained; the formation of such compounds was even used in practice for the sorption of *s*-element ions, in particular Rb^+^ and Cs^+^, from various aqueous solutions [165,166,167,168]. In principle, ion-exchange reactions should also be observed in the interaction of mononuclear (*d*)- or (*f*)-metal hexacyanoferrates(II) with salts of various *d*-elements. However, there is practically no information on the formation of heteronuclear hexacyanoferrates(II) upon contact with solid-phase hexacyanoferrates(II) of cobalt(II), nickel(II), copper(II) and other doubly charged ions of d-elements mentioned above with aqueous solutions of chlorides of other d-elements, in that there is no literature available to us; the only known publication in this regard is [169], where heteronuclear hexacyanoferrates(II) containing (MM’) (CoCu), (NiCu), (ZnCu), and (CdCu) were obtained. There is also no mention of (*dd*)heteronuclear hexacyanoferrates in the review article [170], in which cyano complexes of d-elements were considered as potential compounds for the creation of molecular ferromagnets. However, taking into account that gelatin-immobilized matrix systems are unique nanoreactors and organizing systems, it can be assumed that (*dd*)heteronuclear hexacyanoferrates(II) can in principle arise as a result of ion-exchange reactions occurring upon contact with gelatin-immobilized mononuclear hexacyanoferrates(II) with aqueous solutions containing soluble salts of other *d*-metal ions. Publications [171,172,173,174,175,176,177,178,179,180,181,182,183,184,185] are devoted to studies in this direction. According to the data of these works, ion exchange in metal hexacyanoferrate(II) gelatin-immobilized matrix systems is a very specific phenomenon in the chemistry of cyano-complexes and leads to the formation of very chemically complex coordination compounds, the existence of which researchers have not yet suspected. In total, more than thirty different gelatin-immobilized hexacyanoferrates containing in their composition, in addition to Fe, atoms of such d-elements as Mn, Co, Ni, Cu, Zn, and Cd, were obtained; their preparation and properties are described in detail in [171,172,173,174,175,176,177,178,179,180,181,182,183,184,185]. What is characteristic, in none of the systems [metal hexacyanoferrate(II) gelatin-immobilized matrix—*d*-element chloride] was a complete replacement of the metal ion contained in this matrix by the metal ion contained in the solution in contact with it; this circumstance may serve as an indication of the higher thermodynamic stability of heteronuclear hexacyanoferrates(II) compared to the initial mononuclear ones. It should also be noted that the inversion of the substituted and substituting *d*-metal ions also changes the stoichiometric composition of the resulting heteronuclear hexacyanoferrates; so, in the system Co_2_[Fe(CN)_6_]—CuCl_2_, Co_2_Cu_14_[Fe(CN)6]_8_ is formed, while in the system Cu_2_[Fe(CN)_6_]—CoCl_2_—Cu_11_Co_5_[Fe(CN)_6_]_8_ is formed [171,178]. The unusual chemical composition of these compounds once again emphasizes the decisive role in the formation of (*dd*)heteronuclear hexacyanoferrates(II) of the gelatin matrix, which in this case plays the role of an organizing system. It is very likely that a similar behavior with respect to electrophilic substitution reactions will also take place in the case of other mononuclear hexacyanoferrates(II), as well as the above heteronuclear hexacyanoferrates(II) (in the latter case, metal hexacyanoferrates(II) containing three different *d*-elements may well be formed), but this question is still open. There is no information about the sizes and shapes of particles of gelatin-immobilized metal hexacyanoferrates(II) in the literature yet, but taking into account what was said above in Section 2, Section 3, Section 4 and Section 5, there is every reason to believe that they will also have a nanostructural level of organization. There are also review articles on this subject [186,187].

## 7. Possible Applications of Gelatin-Immobilized Systems Formed by Metal-Containing Chemical Compounds

In general, the objects obtained as a result of the introduction of various substances into the gelatin matrix are rather widely used, primarily in the field of medicine and pharmacology, where they are used as drug carriers. As mentioned in the Introduction, the immobilization of a substance in a gelatin matrix can be carried out either by dispersing pre-created particles of a given substance in an array or a thin layer of gelatin or by forming particles of a given substance directly in an array or a thin layer of a given biopolymer as a result of chemical transformations occurring with the participation of other gelatin-immobilized chemical compounds. At present, the first of these variants is mainly used in practice, while the second one remains relatively less in demand (although it seems to be more promising). In connection with this variant, special mention should be made of the application of metal-containing gelatin-immobilized systems in medical practice, where in recent years there has been a clear trend towards the use of targeted drug delivery based on gelatin modified with various functional groups, as well as composite materials with embedded gelatin carriers. Firstly, gelatin is a natural compound, and therefore, a priori, it seems to be a very convenient medium for creating and transporting various drugs into the human body. Secondly, it is a hydrophilic high-molecular compound and one of those protein products that are produced during human life. Thirdly, both by itself and in combination with water, it easily forms very strong jellies, into which both macro-, micro- and nanoparticles of various substances can be implanted.

A very significant portion of the gelatin used in medicine and the related pharmaceutical industry is sold for the manufacture of hard and soft gelatin capsules (soft gels), as well as for tableting, tablet coating, granulation, encapsulation and microencapsulation. Encapsulation of drugs seems to be very appropriate in cases where it is required to eliminate or minimize the bad (bitter) taste and/or smell of drugs, and protect them from exposure to light or aggressive environmental agents (in particular, atmospheric oxygen) [188]. Because of this, the controlled and/or directed release of bioactive molecules contained in gelatin capsules is also made possible [189,190]. For the encapsulation of drugs in such capsules, it may be useful to cross-link gelatin polymer chains in order, on the one hand, to help reduce solubility in body fluids, and on the other hand, to provide a prolonged release of the encapsulated drug [191,192]. Gelatin-immobilized substances in such systems usually consist of microparticles, although, in principle, nanoparticles can also appear in them. A number of publications are devoted to research in this direction, where gelatin-immobilized systems are proposed for transporting drugs, containing, along with gelatin, some other substances—natural and synthetic, organic and inorganic. Among the publications that considered the possibility of using metal-containing gelatin-immobilized systems are, in particular, [188,189,190,191,192,193,194,195,196,197,198,199,200,201,202,203,204], in which hydroxyapatite Ca_5_(PO_4_)_3_(OH) [193,194,195,196,197,198,199] was used as an immobilized substance (which was first described in this capacity, a minimum of 20 years ago [188]) and trisubstituted calcium orthophosphate Ca_3_(PO_4_)_2_, which is close to it in composition [200,201,202,203,204]. The problem of drug encapsulation is closely related to a very important task from a practical point of view—targeted delivery of drugs to tissues and internal organs; its solution is of particular importance when it comes to the diagnosis and treatment of oncological diseases [205,206]. It is quite obvious that such a targeted delivery to a specific tissue/organ, on the one hand, provides higher therapeutic efficacy of any drug, on the other hand, significantly reduces side effects and general toxicity to the human body, which to some extent occurs with its use. The use of gelatin as a component of systems for targeted delivery seems to be a very interesting and promising direction in the physical chemistry of drugs, and a very significant number of publications are devoted to research in this direction. However, it cannot be ignored, that the range of drugs based on metal-containing compounds in medical practice is still very limited, largely because metal-containing compounds, with rare exceptions, are xenobiotics (although it has long been known that the biological activity of at least those drugs which are able to form complexes with metal ions, is sharply enhanced in the presence of the corresponding metal ions). That is why the proportion of those works that considered targeted delivery using metal-containing gelatin-immobilized systems, among the entire array of works devoted to targeted delivery using gelatin-based compositions, is quite small. These include mainly publications in which the immobilized substance was the so-called cisplatin (*cis*-diamminedichloroplatinum(II) [Pt(NH_3_)_2_Cl_2_]), which has been widely used in the treatment of various oncological diseases since the 1980s and is one of the most active anticarcinogenic drugs [207,208,209,210]. Recently, a paper was published [211], which showed the possibility of using gelatin to deliver a copper(II) compound with methotrexate. The problems associated with targeted drug delivery are discussed in [212,213] and, also, in very recent reviews [214,215,216]. As for the metal-containing gelatin-immobilized systems obtained in the framework of the second of the above methods, they, unfortunately, have not yet received any serious practical application. Only one area of their application, which, however, is more related to pure science than to practice, namely, to record the absorption spectra of various chemical compounds, and primarily metal-containing ones [217,218], was described in the literature. Quite a long time ago, the possibility of creating light-sensitive materials for holography based on metal-complex gelatin-immobilized systems containing Cr(III) polymer complexes with gelatin and obtained by treating gelatin with solutions containing dichromate anion (Cr_2_O_7_^2–^) was noted. Judging by publications of recent years [219,220,221,222,223], research in this direction has not lost its relevance. The use of gelatin-immobilized systems as biosensors is of great interest; the possibilities in this respect are presented in the reviews [224,225]. In principle, metal-containing gelatin-immobilized systems of both the first and second of the above immobilization options can also be used in other branches of science and technology, for example, in metal complex catalysis, to create liquid crystal systems, specific electrodes for implementing redox processes, and others, but their capabilities in this regard have so far remained undiscovered, and this is probably a matter for the future.

## 8. Conclusions

As can be seen from the data presented in Section 5 and Section 6, using the reactions of nucleophilic, electrophilic substitution and template synthesis, a very significant range of gelatin-immobilized metal-containing chemical compounds, usually consisting of nanoparticles, can be obtained. This assortment can be further expanded without any particular problems by increasing both the number of metal hexacyanoferrate(II) gelatin-immobilized precursors and organic compounds (ligands, ligand synthons, and other nucleophilic agents). However, the synthetic possibilities in gelatin-immobilized matrix systems are by no means exhausted by this, because there are also such options as (a) expanding the range of metal-containing precursors, (b) expanding the range of chemical reactions occurring with the participation of both metal hexacyanoferrate (II) and other metal-containing precursors. Within the framework of option (a), in particular, instead of silver hexacyanoferrate(II), chromates(VI) can be used, which are easily obtained by the oxidation of gelatin-immobilized elemental silver with a solution of potassium dichromate(VI). As already mentioned in Section 6, using electrophilic substitution reactions in metal hexacyanoferrate(II) gelatin-immobilized matrices, heterobinuclear metal hexacyanoferrates(II) can be obtained, any of which, in turn, can also be used as a precursor in nucleophilic substitution reactions; in this case, the possibility opens up for the synthesis of heteronuclear metal-containing compounds (and, first of all, metal chelates). For the immobilization of chemical compounds in a gelatin matrix, in principle, it is also possible to use reagents that are under standard conditions (under which, as a rule, most chemical reactions are carried out) in the gas phase. It should be noted, in this connection, that gelatin-immobilized systems obtained by forming particles of a given substance directly in an array or a thin layer of a given biopolymer as a result of chemical transformations and having a nanoscale level of organization, seem to be more promising for use, and above all for targeted drug delivery to various tissues of the human body, than gelatin-immobilized systems obtained by dispersing particles of substances in an array or thin layer of gelatin (which, as a rule, have a micro-sized level of organization). There is every reason for such a statement since it has long been found that the reactivity of chemical substances consisting of nanoparticles in chemical and biochemical processes (and, consequently, the biological activity associated with it) is much higher than that for the same substances consisting of micro- or macroparticles.

## Data Availability

No unpublished data was created or analyzed in this article.
